# Correction: Wan et al. Correlation between Hypoperfusion Intensity Ratio and Functional Outcome in Large-Vessel Occlusion Acute Ischemic Stroke: Comparison with Multi-Phase CT Angiography. *J. Clin. Med.* 2022, *11*, 5274

**DOI:** 10.3390/jcm12123990

**Published:** 2023-06-12

**Authors:** Zhifang Wan, Zhihua Meng, Shuangcong Xie, Jin Fang, Li Li, Zhensong Chen, Jinwu Liu, Guihua Jiang

**Affiliations:** 1 The Second School of Clinical Medicine, Southern Medical University, Guangzhou 510515, China; 2 The Department of Medical Imaging, Guangdong Second Provincial General Hospital, Guangzhou 510317, China; 3 Department of Medical Image Center, Yuebei People’s Hospital, Shaoguan 512025, China

**Additional Affiliation(s).**

In the published publication [1], there was an error regarding the affiliation(s) for **“Guihua, Jiang”**. In addition to affiliation(s) **“2 The Department of Medical Imaging, Guangdong Second Provincial General Hospital, Guangzhou 510317, China;”**, the updated affiliation(s) should include: **“1 The Second School of Clinical Medicine, Southern Medical University, Guangzhou 510515, China;”**. The authors state that the scientific conclusions are unaffected. This correction was approved by the Academic Editor. The original publication has also been updated.
