# Supplementary material for: Correlation between Hypoperfusion Intensity Ratio and Functional Outcome in Large-Vessel Occlusion Acute Ischemic Stroke: Comparison with Multi-Phase CT Angiography
Source: J Clin Med. 2022 Sep 7;11(18):5274. doi: 10.3390/jcm11185274 (PMC9503156; doi:10.3390/jcm11185274)
Supplement: Supplementary file 1 [file jcm-11-05274-s001.zip › jcm-1844473-supplementary.pdf]

Journal of Clinical Medicine

MDPI

Supplementary Material

1 Supplementary Tables

Supplementary Table S1. Baseline Patient Characteristics in reperfusion therapy group and Comparison of Clinical Variables Stratified by Functional Outcome

No. of patients and characteristics	ALL<br>n= 118(100%)	Favorable outcome<br>n = 70(59%)	Unfavorable outcome<br>n =48(41%)	*P*-value
**Age, Median (IQR)**	**66 [55.2, 73]**	**64.5 [55, 72]**	**68.5 [56, 74.2]**	0.275
**Male, n (%)**	**94 (79.7)**	**58 (82.9)**	**36 (75)**	0.419
**Risk factors n (%)**				
**Hypertension, n (%)**	**76 (64.4)**	**44 (62.9)**	**32 (66.7)**	0.819
**Diabetes, n (%)**	**27 (22.9)**	**10 (14.3)**	**17 (35.4)**	0.014
**Hyperlipidemia, n (%)**	**38 (32.2)**	**25 (35.7)**	**13 (27.1)**	0.432
**Prior stroke, n (%)**	**23 (19.5)**	**13 (18.6)**	**10 (20.8)**	0.946
**Coronary artery disease, n (%)**	**21 (17.8)**	**13 (18.6)**	**8 (16.7)**	0.983
**Valvular disease, n (%)**	**23 (19.5)**	**10 (14.3)**	**13 (27.1)**	0.137
**Chronic heart failure, n (%)**	**7 (5.9)**	**3 (4.3)**	**4 (8.3)**	0.44
**Atrial fibrillation, n (%)**	**26 (22)**	**10 (14.3)**	**16 (33.3)**	**0.026**
**Smoke, n (%)**	**42 (35.6)**	**27 (38.6)**	**15 (31.2)**	0.535
**Drink, n (%)**	**22 (18.6)**	**16 (22.9)**	**6 (12.5)**	0.239
**Homocysteine, Median [IQR]**	**12.8 [10, 16.5]**	**12.2 [10, 16.2]**	**13.1 [10.1, 17]**	0.35
**SBP, Median [IQR]**	**143.5 [129, 162]**	**145.5[128, 162]**	**140[130, 160]**	0.724
**DBP, Median [IQR]**	**85 [76, 96]**	**86 [76, 98]**	**82.5 [74, 90]**	0.348
**HR, Median [IQR]**	**80 [71, 88]**	**79 [70, 86]**	**81 [75, 96]**	**0.16**
**Blood Glucose, Median [IQR]**	**5.8 [5.2, 7.2]**	**5.6 [4.8, 6.7]**	**6.4 [5.7, 8.5]**	**< 0.001**
**Glycosylated hemoglobin, Median[IQR]**	**5.8 [5.5, 6]**	**5.7 [5.4, 5.9]**	**5.9 [5.6, 6.5]**	0.007
**Time to CT(min) ,Median[IQR]**	**480[276, 744]**	**480[270, 744]**	**480[300, 732]**	0.943
**Baseline NIHSS, Median[IQR]**	**11[7, 17]**	**8[6, 14]**	**15 [10, 19]**	**< 0.001**
**Imaging items**				
**ASPECTS on NCCT, Median[IQR]**	**7[6, 8]**	**8[7, 8]**	**6[5, 7]**	**< 0.001**
**Location of occlusion on mCTA, n (%)**				0.899
**ICA or Tandem**	**39 (33.1)**	**24 (34.3)**	**15 (31.2)**	
**M1**	**56 (47.5)**	**32 (45.7)**	**24 (50)**	
**M2 or further distal**	**23 (19.5)**	**14 (20)**	**9 (18.8)**	
**mCTA score, n (%)**				**0.003**
**1**	**4 (3.4)**	**0 (0)**	**4 (8.3)**	
**2**	**8 (6.8)**	**2 (2.9)**	**6 (12.5)**	
**3**	**64 (54.2)**	**36 (51.4)**	**28 (58.3)**	
**4**	**35 (29.7)**	**27 (38.6)**	**8 (16.7)**	
**5**	**7 (5.9)**	**5 (7.1)**	**2 (4.2)**	
**Good collaterals (4-5)**	**42(35.6)**	**32(45.7)**	**10(20. 8)**	
**Poor collaterals (1-3)**	**76(64.4)**	**38(54.3)**	**38(79.2)**	
**mCTA score , Median [IQR]**	**3[3, 4]**	**3[3, 4]**	**3[2, 3]**	**< 0.001**
**ischemic core volume(rCBF<30%) (mL), Median [IQR]**	**4.5[1.8, 15.4]**	**2.2[1.1, 5.5]**	**12.6[5.2, 30.4]**	**< 0.001**
**Mismatch ratio, Median [IQR]**	**15.8[6, 40.4]**	**23.1[11.7, 55.8]**	**7[4.3, 19.1]**	**< 0.001**
**TMax>6s volume(mL), Median [IQR]**	**82.6[55.4, 126.4]**	**70.2[43.4, 97.8]**	**99.8[72.4, 167.8]**	**< 0.001**
**TMax>10s volume(mL), Median [IQR]**	**18.2[6.8, 37.6]**	**7.7[4.4, 17.5]**	**38.8[28.2, 67]**	**< 0.001**
**HIR, Median [IQR]**	**0.2[0.1, 0.4]**	**0.1[0.1, 0.2]**	**0.4[0.3, 0.4]**	**< 0.001**
**FIV, Median [IQR]**	**20.6[8.6, 73.2]**	**12.1[7.4, 17.6]**	**87[43.4, 166]**	**< 0.001**
**Type of treatment, n (%)**				0.358
**Intravenous thrombolysis**	**31 (26.3)**	**19 (27.1)**	**12 (25)**	
**Bridging therapy**	**14 (11.9)**	**7 (10)**	**7 (14.6)**	
**EVT**	**73 (61.9)**	**44 (62.9)**	**29 (60.4)**	
**90d_mRS, Median [IQR]**	**2[1, 4]**	**1[0, 2]**	**4[3, 5]**	**< 0.001**

Supplementary Table S2. Baseline Patient Characteristics in IVT and EVT group and Comparison of Clinical Variables Stratified by Functional Outcome

No. of patients and characteristics	IVT			EVT		
	Favorable outcome n = 19(61.3%)	Unfavorable outcome n =12(38.7%)	*P*-value	Favorable outcome n = 51(58.6%)	Unfavorable outcome n =36(41.4%)	*P*-value
**Age**	**62[58.5, 71]**	**69.5 [54, 75]**	0.405	**66[51.5, 72]**	**68 [56, 74]**	0.41
**Male, n (%)**	**16 (84.2)**	**7 (58.3)**	0.206	**42 (82.4)**	**29 (80.6)**	1
**Blood Glucose**	**5.2[4.6, 5.7]**	**6.1 [5.0, 6.9]**	**0.054**	**5.8[5.1, 7.3]**	**6.5[5.8, 8.6]**	**0.008**
**Time to CT(min)**	**240[180, 372]**	**288[150, 300]**	0.568	**570[360, 870]**	**648[414, 780]**	0.746
**Baseline NIHSS**	**7[5, 10]**	**15 [9, 17]**	**0.003**	**10[7, 16]**	**16 [10, 20]**	**0.003**
**ASPECTS**	**8[8, 8]**	**6[6, 7]**	**< 0.001**	**7[7, 8]**	**6[5, 7]**	**< 0.001**
**Location of occlusion**			0.053			0.246
**ICA or Tandem**	**9(47.4)**	**3 (25)**		**15(29.4)**	**12 (33.3)**	
**M1**	**9(47.4)**	**4 (33.3)**		**23(45.1)**	**20 (55.6)**	
**M2**	**1(5.3)**	**5(41.7)**		**13(25.5)**	**4(11.1)**	
**mCTA score, n (%)**			**0.04**			**0.037**
**1**	**0 (0)**	**1(8.3)**		**0 (0)**	**3(8.3)**	
**2**	**0 (0)**	**0(0)**		**2 (3.9)**	**6(16.7)**	
**3**	**7 (36.8)**	**9 (75)**		**29 (56.9)**	**19 (52.8)**	
**4**	**9 (47.4)**	**1(8.3)**		**18 (35.3)**	**7(19.4)**	
**5**	**3(15.8)**	**1 (8.3)**		**2(3.9)**	**1 (2.8)**	
**Good collaterals(4-5)**	**12(63.2)**	**2(16.7)**		**20(39.2)**	**8(22.2)**	
**Poor collaterals(1-3)**	**7(36.8)**	**10(83.3)**		**31(60.8)**	**28(77.8)**	
**mCTA score**	**4[3, 4]**	**3[3, 3]**	**0.043**	**4[3, 4]**	**3[3, 3]**	**0.04**
**rCBF<30%)(mL)**	**1.6[0.6, 3.8]**	**11.2[6.0, 28.3]**	**< 0.001**	**2.5[1.2, 6.5]**	**12.9[5.2, 30.4]**	**< 0.001**
**Mismatch ratio**	**17[13.1,26.9]**	**7.6[3, 15.9]**	**0.024**	**27.6[10.8,58.9]**	**7.0[4.4, 19.1]**	**< 0.001**
**TMax>6s (mL)**	**29.2[19.5, 72.3]**	**70.8[66.1, 95.8]**	**0.012**	**79.4[55.8, 114]**	**117.9[84, 184.7]**	**0.001**
**TMax>10s (mL)**	**3.8[2, 8.8]**	**30.8[26.4, 37.1]**	**< 0.001**	**8.9[5.5, 21.2]**	**40[31.3, 78.2]**	**< 0.001**
**HIR**	**0.2[0.1, 0.2]**	**0.4[0.4, 0.5]**	**< 0.001**	**0.1[0.1, 0.2]**	**0.4[0.3, 0.4]**	**< 0.001**
**FIV**	**8.4[5.8, 16.1]**	**74.7[56.2, 142.9]**	**< 0.001**	**12.8[8, 22.4]**	**99.2[36.6, 169.2]**	**< 0.001**
**90d_mRS**	**1[0, 1]**	**4[3, 4]**	**< 0.001**	**1[0, 2]**	**4[3, 5]**	**< 0.001**

Values in parentheses represent percentage of patients (%); brackets represent first and third quartiles, respectively.

Abbreviations: NIHSS, National Institutes of Health Stroke Scale; ASPECTS, Alberta Stroke Program Early Computed Tomography Score; mCTA, multiphase CTA; rCBF, relative cerebral blood flow; TMax, the time when the residue function reaches its maximum; HIR, hypoperfusion intensity ratio.

Supplementary Table S3. Baseline Patient Characteristics in supportive medical treatment group and Comparison of Clinical Variables Stratified by Functional Outcome

No. of patients and characteristics	ALL n= 117(100%)	Favorable outcome n = 57(48.7%)	Unfavorable outcome n =60(51.3%)	*P*-value
**Age, Median (IQR)**	**65 [56, 72]**	**65 [55, 70]**	**65.5 [56.8, 74.2]**	0.606
**Male, n (%)**	**84 (71.8)**	**45 (78.9)**	**39 (65)**	0.142
**Risk factors n (%)**				
**Hypertension, n (%)**	**99 (84.6)**	**46 (80.7)**	**53 (88.3)**	0.375
**Diabetes, n (%)**	**34 (29.1)**	**16 (28.1)**	**18 (30)**	0.979
**Hyperlipidemia, n (%)**	**55 (47)**	**27 (47.4)**	**28 (46.7)**	1
**Prior stroke, n (%)**	**30 (25.6)**	**13 (22.8)**	**17 (28.3)**	0.637
**Coronary artery disease, n (%)**	**21 (17.9)**	**14 (24.6)**	**7 (11.7)**	0.115
**Valvular disease, n (%)**	**20(17.1)**	**7 (12.3)**	**13 (21.7)**	0.27
**Chronic heart failure, n (%)**	**13 (11.1)**	**4 (7)**	**9 (15)**	0.281
**Atrial fibrillation, n (%)**	**21 (17.9)**	**7 (12.3)**	**14 (23.3)**	**0.188**
**Smoke, n (%)**	**40 (34.2)**	**23 (40.4)**	**17 (28.3)**	0.24
**Drink, n (%)**	**14 (12)**	**6 (10.5)**	**8 (13.3)**	0.855
**Homocysteine, Median [IQR]**	**13.3 [10.8, 16.3]**	**13.3[10.8, 16.2]**	**13.3 [10.9, 16.6]**	0.952
**SBP, Median [IQR]**	**150 [133, 167]**	**142[130, 169]**	**152[137.5, 164]**	0.39
**DBP, Median [IQR]**	**89 [80, 96]**	**88 [80, 95]**	**89 [80,99]**	0.466
**HR, Median [IQR]**	**78 [74, 90]**	**78 [72, 86]**	**80 [74, 97]**	**0.09**
**Blood Glucose, Median [IQR]**	**6 [5.2, 8.1]**	**5.6 [5.1, 7.1]**	**6.6 [5.7, 8.5]**	**0.037**
**Glycosylated hemoglobin, Median[IQR]**	**5.9[5.6, 6.4]**	**5.9 [5.7, 6.5]**	**5.8 [5.4, 6.4]**	0.051
**Time to CT(min) ,Median[IQR]**	**690[450, 1020]**	**630[420, 900]**	**750[474, 1080]**	0.114
**Baseline NIHSS, Median[IQR]**	**10[4, 15]**	**5[3, 10]**	**14 [8, 19]**	**< 0.001**
**Imaging items**				
**ASPECTS on NCCT, Median[IQR]**	**7[6, 7]**	**7[7, 8]**	**6[4, 7]**	**< 0.001**
**Location of occlusion on mCTA, n (%)**				0.414
**ICA or Tandem**	**44 (37.6)**	**21 (36.8)**	**23 (38.3)**	
**M1**	**48 (41)**	**21 (36.8)**	**27 (45)**	
**M2 or further distal**	**25(21.4)**	**15 (26.3)**	**10 (16.7)**	
**mCTA score, n (%)**				**< 0.001**
**1**	**16 (13.7)**	**0 (0)**	**16 (26.7)**	
**2**	**9 (7.7)**	**0 (0)**	**9 (15)**	
**3**	**53 (45.3)**	**27 (47.4)**	**26 (43.3)**	
**4**	**28 (23.9)**	**19 (33.3)**	**9 (15)**	
**5**	**11 (9.4)**	**11 (19.3)**	**0 (0)**	
**Good collaterals (4-5)**	**39(33.3)**	**30(52.6)**	**9(15)**	
**Poor collaterals (1-3)**	**78(66.7)**	**27(47.4)**	**51(85)**	
**mCTA score , Median [IQR]**	**3[3, 4]**	**3[3, 4]**	**3[2, 3]**	**< 0.001**
**ischemic core volume(rCBF<30%)(mL), Median [IQR]**	**4.8[1.7, 26.9]**	**1.8[0.9, 3.4]**	**16.9[6.4, 54.7]**	**< 0.001**
**Mismatch ratio, Median [IQR]**	**10.8[3.3, 28.1]**	**22.9[12.2, 39.8]**	**3.8[1.7, 11.3]**	**< 0.001**
**TMax>6s volume(mL), Median [IQR]**	**66.8[38.7, 117.9]**	**42.8[27.4, 66.8]**	**95.7[66.3, 138.9]**	**< 0.001**
**TMax>10s volume(mL), Median [IQR]**	**17.5[5.3, 41.7]**	**5.3[2.8, 12.2]**	**41.2[23.6, 63.4]**	**< 0.001**
**HIR, Median [IQR]**	**0.3[0.1, 0.4]**	**0.2[0.1, 0.2]**	**0.4[0.4, 0.5]**	**< 0.001**
**FIV, Median [IQR]**	**29.3[12.6, 78.4]**	**13.8[7.9, 20]**	**77.3[45.2, 156.3]**	**< 0.001**
**90d_mRS, Median [IQR]**	**3[2, 4]**	**2[1, 2]**	**4[3, 4]**	**< 0.001**

Values in parentheses represent percentage of patients (%); brackets represent first and third quartiles, respectively.

Abbreviations: SBP, systolic pressure; DBP, diastolic blood pressure; HR, heart rate; NIHSS, National Institutes of Health Stroke Scale; ASPECTS, Alberta Stroke Program Early Computed Tomography Score; NCCT, unenhanced CT; mCTA, multiphase CTA;ICA, internal cerebral artery; rCBF, relative cerebral blood flow; TMax, the time when the residue function reaches its maximum; HIR, hypoperfusion intensity ratio; FIV, follow-up infarct volume; EVT, endovascular thrombectomy; mRS, modified Rankin scale.

Supplementary Table S4.Multivariable logistic regression analysis for functional outcome in different type of treatment arm

Variables	reperfusion treatment group (n=118) aOR (95% CI)	*p*-value	Supportive medical treatment group(n=117) aOR (95% CI)	*p*-value
**Age**	1 (0.91-1.09)	0.924	1 (0.92-1.09)	0.935
**Gender**	0.9 (0.05-15.78)	0.944	0.34 (0.03-3.91)	0.386
**Blood Glucose**	1.48 (0.99-2.23)	0.058	1.12 (0.78-1.6)	0.547
**NIHSS**	1.18 (0.98-1.43)	0.086	1.05 (0.86-1.28)	0.616
**ASPECTS**	0.58 (0.18-1.9)	0.37	0.27 (0.06-1.1)	0.068
**mCTA score**	0.2 (0.03-1.48)	0.117	0.48 (0.11-2.22)	0.349
**rCBF<30%**	0.93 (0.87-1)	0.054	1 (0.91-1.09)	0.914
**TMax > 6s**	1 (0.99-1.02)	0.903	1 (0.98-1.02)	0.949
**HIR（per 0.01）**	1.55 (1.21-1.98)	**<0.001**	1.31 (1.14-1.5)	**<0.001**

we adjusted age, Gender , blood glucose, NIHSS,ASPECTS, mCTA score, rCBF<30% , TMax > 6s

Abbreviations: NIHSS, National Institutes of Health Stroke Scale; ASPECTS, Alberta Stroke Program Early Computed Tomography Score; mCTA, multiphase CTA; rCBF, relative cerebral blood flow; TMax, the time when the residue function reaches its maximum; HIR, hypoperfusion intensity ratio.

Supplementary Table S5. Multivariable logistic regression analysis for functional outcome in EVT group

Variables	EVT group(n=87)			
	Crude OR, 95%CI	*p*-value	aOR, 95%CI	*p*-value
**Age**	1.01 (0.98-1.05)	0.44		
**Gender**	1.13 (0.38-3.37)	0.831		
**Blood Glucose**	1.12 (0.96-1.3)	0.137		
**NIHSS**	1.11 (1.03-1.19)	**0.004**	1.14 (0.96-1.34)	0.13
**ASPECTS**	0.47 (0.3-0.71)	**<0.001**	0.65 (0.21-2.04)	0.457
**mCTA score**	0.42 (0.22-0.81)	**0.009**	0.21 (0.03-1.51)	0.12
**rCBF<30%**	1.05(1.01-1.08)	**0.006**	0.97 (0.92-1.01)	0.166
**TMax > 6s**	1.01 (1 -1.02)	**0.006**	0.94 (0.87-1.01)	0.083
**HIR（per 0.01）**	1.32 (1.17-1.48)	**<0.001**	1.49 (1.18-1.88)	**<0.001**

Crude model: no other covariates were adjusted.

Adjusted model: we adjusted NIHSS, ASPECTS, mCTA score, rCBF<30% , TMax > 6s

Supplementary Table S6. Correlation between HIR , mCTA with Functional Outcome in different subgroup

Variable	Functional Outcome(90-d mRS)									
	Total (n=235)	*p*-value	group 1 (n=118)	*p*-value	group 2 (n=117)	*p*-value	group 3 (n=117)	*p*-value	group 4 (n=117)	*p*-value
**HIR**	0.8519	*P*<0.0001	0.8413	*P*<0.0001	0.8801	*P*<0.0001	0.8714	*P*<0.0001	0.8477	*P*<0.0001
**mCTA**	- 0.4657	*P*<0.0001	- 0.3269	*P*=0.0003	- 0.6049	*P*<0.0001	- 0.4561	*P*=0.0003	- 0.466	*P*<0.0001

group 1 reperfusion treatment; group 2 Supportive medical treatment; group 3 early time window(<6h); group 4 late time window(6-24h)
